# MARCH5 Links Hypoxia to Neutrophil Ferroptosis in SLE via FSP1 Ubiquitination

**DOI:** 10.1002/iid3.70583

**Published:** 2026-11-10

**Authors:** Jing Zhang, Fulin Cai, Jiansen Lu, Zhiwei Liu

**Affiliations:** ^1^ Department of Blood Transfusion, School of Medicine, Sir Run Run Shaw Hospital Zhejiang University Hangzhou Zhejiang China; ^2^ The First Hospital of Anhui University of Science and Technology Huainan China; ^3^ Department of Orthopaedics, Shaoguan First People's Hospital Affiliated Hospital of Shaoguan University Shaoguan China

**Keywords:** ferroptosis, FSP1, hypoxia, MARCH5, SLE

## Abstract

**Purpose:**

Ferroptosis plays an important role in the progression of systemic lupus erythematosus (SLE). This study aims to investigate the molecular mechanism by which membrane‐associated RING‐CH‐type finger protein 5 (MARCH5) regulates ferroptosis suppressor protein 1 (FSP1), a key regulator of ferroptosis resistance, under hypoxic conditions and its impact on SLE progression.

**Methods:**

We established a Toll‐like receptor 7/8 (TLR7/8) agonist‐induced mouse model of SLE under normoxic or hypoxic conditions and longitudinally evaluated pathological manifestations and survival. In vitro, the effects of hypoxia on ferroptosis induced by glutathione peroxidase 4 (GPX4) inhibitors or FSP1 inhibitors were assessed using the Cell Counting Kit‐8 (CCK‐8) assay. Cycloheximide (CHX) chase assays and pharmacological inhibitors of protein degradation pathways were employed to investigate how hypoxia regulates FSP1 stability. Candidate E3 ligases involved in FSP1 ubiquitination were identified using bioinformatic prediction tools and validated through *March5* knockdown or overexpression experiments. Finally, neutrophil‐specific *March5* knockout mice were generated to further demonstrate that hypoxia promotes SLE progression through a MARCH5‐dependent mechanism.

**Results:**

Chronic hypoxia significantly exacerbates SLE pathology by selectively disrupting FSP1‐dependent, rather than GPX4‐dependent, ferroptosis resistance in neutrophils. Mechanistically, hypoxia does not affect *Aifm2* (i.e., the gene encoding FSP1) mRNA expression but accelerates FSP1 protein degradation through MARCH5‐mediated ubiquitination and proteasomal turnover. Hypoxia enhances the interaction between MARCH5 and FSP1, promoting FSP1 destabilization. Knockdown of MARCH5 prevents hypoxia‐induced FSP1 degradation and neutrophil ferroptosis, whereas neutrophil‐specific *March5* deletion improves survival, reduces autoantibody production, restores complement levels, and alleviates organ damage in SLE mice.

**Conclusions:**

This study reveals a pathogenic axis whereby hypoxia compromises neutrophil ferroptosis resistance and exacerbates SLE progression through MARCH5‐mediated ubiquitination and degradation of FSP1. These findings elucidate how impaired FSP1‐dependent ferroptosis defense contributes to chronic inflammatory disease progression and identify the MARCH5–FSP1 interaction as a potential therapeutic target for SLE and other ferroptosis‐associated autoimmune disorders.

## 1 Introduction

Systemic lupus erythematosus (SLE) is a chronic autoimmune disease characterized by profound immune system dysfunction and heterogeneous clinical manifestations that may involve multiple organ systems, including the kidneys (lupus nephritis), skin (malar rash, photosensitivity), neuropsychiatric system (seizures, psychosis), and cardiovascular system (pericarditis, atherosclerosis) [1, 2, 3]. Hallmark pathological features of SLE include elevated type I interferon (IFN‐I) signaling, excessive autoantibody production, and accumulation of uncleared cellular debris derived from various forms of cell death, including apoptosis, necrosis, NETosis, and ferroptosis [4, 5, 6, 7, 8]. These interconnected processes establish a self‐amplifying inflammatory circuit that drives disease progression and heterogeneity.

Ferroptosis is an iron‐dependent form of regulated cell death characterized by excessive membrane lipid peroxidation [9]. In SLE, iron accumulation, oxidative stress, and lipid peroxidation are prominent pathological features and potential triggers of ferroptosis. Ferroptotic cells can influence autoimmune responses by directly altering immune cell viability and function or by releasing damage‐associated signals that activate inflammatory pathways [10, 11, 12]. Neutrophils, the most abundant circulating immune cells, play critical roles in SLE pathogenesis, and dysregulated neutrophil ferroptosis has been implicated in neutrophil loss and disease progression [8, 13].

Ferroptosis suppressor protein 1 (FSP1) represents a GPX4‐independent ferroptosis defense pathway and functions primarily through the FSP1–coenzyme Q10 (CoQ10)–nicotinamide adenine dinucleotide (phosphate) (NAD(P)H) axis and the vitamin K redox cycle [14]. FSP1 activity is regulated by multiple mechanisms, including transcriptional regulation, non‐coding RNAs, and epigenetic modifications, which contribute to ferroptosis‐associated disease processes [15, 16, 17]. Elevated FSP1 expression is closely associated with poor prognosis in malignant tumors and plays a significant role in cancer treatment [18, 19]. Beyond cancer, emerging evidence suggests that FSP1 also contributes to inflammatory and fibrotic pathologies. In inflammatory arthritis, trained macrophages promote an interleukin‐1β (IL‐1β)–N‐acetyltransferase 10 (NAT10)–FSP1 mRNA N4‐acetylcytidine (ac4C) axis in fibroblast‐like synoviocytes, increasing their resistance to ferroptosis and contributing to disease progression [20]. In pulmonary fibrosis, UHRF1‐mediated epigenetic repression of GPX4 and FSP1 promotes ferroptosis in alveolar epithelial cells and exacerbates fibrosis [21]. However, despite evidence that GPX4 regulates neutrophil ferroptosis and protects against SLE, whether and how FSP1 contributes to SLE pathogenesis remains unclear.

Here, we identify a previously unrecognized mechanism regulating FSP1 in SLE. We demonstrate that hypoxia promotes MARCH5‐mediated ubiquitination and proteasomal degradation of FSP1, thereby impairing neutrophil ferroptosis defense and exacerbating SLE progression. These findings establish a hypoxia–MARCH5–FSP1 axis in autoimmune pathogenesis and suggest that targeting this pathway may represent a potential therapeutic strategy for SLE.

## 2 Methods and Materials

### 2.1 Animal Models and Hypoxia Exposure

Wild‐type female C57BL/6 mice (8–10 weeks old) were purchased from GemPharmatech (Nanjing, China) and used to establish the systemic lupus erythematosus (SLE) model. Mice were housed under specific pathogen‐free (SPF) conditions at 25°C and a relative humidity of 50% ± 10%, with a 12‐h light/12‐h dark cycle and free access to standard chow and water. Mice were acclimatized to the animal facility for 7 days before experimentation.

SLE was induced by topical application of resiquimod (R848; Cat. No. HY‐13740, MedChemExpress) at 1.5 mg/kg body weight to the right ear three times per week for 5 weeks. R848 was dissolved in a mixture of ethanol and acetone (1:3, v/v). Vehicle‐control mice received the same ethanol/acetone mixture without R848 [22].

Immediately after the first R848 application, mice were randomly assigned to normoxic (21% O_2_) or hypoxic (11% O_2_) conditions in a controlled environmental chamber. Oxygen concentrations were continuously monitored throughout the experimental period. Mice were maintained under the designated oxygen conditions according to the experimental schedule, and survival and clinical parameters were evaluated at the indicated time points. Depending on the specific experimental objectives, mice were either monitored for survival or euthanized at predefined time points for tissue collection. Mice designated for tissue collection were euthanized by cervical dislocation, and the relevant tissues were collected for subsequent analyses. Animals reaching the humane endpoints specified in the approved animal protocol were euthanized accordingly. The timing of tissue collection and the duration of hypoxia exposure for each experiment are specified in the corresponding figure legends.

### 2.2 Generation of Neutrophil‐Specific *March5* Knockout Mice

To generate a neutrophil‐specific deletion of *March5*, mice possessing floxed *March5* alleles (*March5* ^fl/fl^) were crossed with Ly6g‐Cre transgenic mice (Cat. #NM‐KI‐200218, ‌Shanghai Model Organisms Center) expressing Cre recombinase under the control of a Ly6g‐specific promoter. The resulting *March5* ^fl/fl^‐Ly6g‐Cre^+^ offspring were used as the neutrophil‐specific *March5* knockout strain. Genotyping was performed by PCR analysis of genomic DNA isolated from tail biopsies. The efficiency of *March5* deletion in neutrophils was confirmed by quantitative PCR (qPCR) and Western blot analysis.

### 2.3 Isolation of Bone Marrow‐Derived Neutrophils and Treatment

Bone marrow‐derived neutrophils were isolated from the femurs and tibias of 8–12‐week‐old mice. Bone marrow cells were flushed using Roswell Park Memorial Institute (RPMI) 1640 medium (Cat: #10‐040‐CVRC, CORNING), and neutrophils were purified by density gradient centrifugation using Histopaque‐1077 and Histopaque‐1119 (Cat. #10771 and #11191, Sigma‐Aldrich) according to the manufacturer's instructions. Cell purity and viability were assessed by flow cytometry using PE anti‐mouse Ly‐6G (Cat: #127607, BioLegend) and APC anti‐mouse/human CD11b (Cat: #101211, BioLegend) antibodies, together with 7‐AAD staining (Cat: #559925, BD Biosciences), respectively. Neutrophils were defined as the Ly6G^+^CD11b^+^ population, with a typical purity of greater than 90% after isolation. Purified neutrophils were cultured in RPMI 1640 medium supplemented with 10% fetal bovine serum (FBS) and 1% penicillin–streptomycin at 37°C in a humidified atmosphere containing 5% CO_2_. For drug‐treatment experiments, the indicated compounds were added immediately before the cells were placed under normoxic (21% O_2_) or hypoxic (1% O_2_) conditions; cells were not pre‐equilibrated under hypoxia before drug treatment. Cells were maintained under the assigned oxygen condition throughout drug treatment and until harvest. Hypoxic exposures were performed in a controlled hypoxia workstation, and oxygen concentrations were monitored in real time to ensure stable conditions. The exposure duration for each experiment is specified in the corresponding figure legend.

### 2.4 Reagents and Chemical Treatments

The following reagents were used: Erastin (Cat: #HY‐15763, MCE), RSL3 (Cat: #HY‐100218A, MCE), FSEN1 (Cat: #HY‐100579, MCE), cycloheximide (Cat: #46401, Sigma‐Aldrich), MG132 (Cat: #C2211, Sigma‐Aldrich), 3‐methyladenine (3‐MA; Cat: #M9281, Sigma‐Aldrich), and bafilomycin A1 (BafA1; Cat: #B1793, Sigma‐Aldrich). All reagents were dissolved in dimethyl sulfoxide (DMSO) or sterile phosphate‐buffered saline (PBS) as appropriate.

### 2.5 Cell Viability Assay (CCK‐8)

Cell viability was assessed using the Cell Counting Kit‐8 (CCK‐8) assay (Cat: #CK04, Dojindo Laboratories). Neutrophils were seeded at a density of 2 × 10^4^ cells per well in 96‐well plates and treated with the indicated compounds under normoxic or hypoxic conditions. At the end of the treatment, 10 μL of CCK‐8 solution was added to each well, followed by incubation for 1–4 h at 37°C. Absorbance was measured at 450 nm using a microplate reader. Cell viability was calculated relative to untreated control cells.

### 2.6 Assessment of Ferroptosis Markers

Intracellular lipid reactive oxygen species (Lipid ROS) levels were assessed using the fluorescent probe C11‐BODIPY 581/591 (Cat: #D3861, Thermo Fisher Scientific). Neutrophils were incubated with 2 μM C11‐BODIPY for 30 min at 37°C in the dark, washed twice with PBS, and analyzed by flow cytometry. The shift from red to green fluorescence indicated lipid peroxidation. For the detection of 4‐hydroxynonenal (4‐HNE), cells were fixed, permeabilized, and stained with the PE Rabbit anti‐4‐Hydroxynonenal/4‐HNE antibody (Cat: #A26084, Abclonal), followed by the appropriate fluorescent secondary antibody, and analyzed by flow cytometry.

### 2.7 Protein Half‐Life Tracking Assay (CHX Chase)

To determine the half‐life of FSP1, neutrophils were treated with 50 μg/mL cycloheximide (CHX) (Cat: #HY‐12320, MCE) to inhibit new protein synthesis. Cells were collected at the indicated time points after CHX treatment, and whole‐cell lysates were subjected to Western blot analysis. Relative protein levels were quantified using densitometry and normalized to the loading control. Protein half‐life was calculated by fitting the data to a first‐order exponential decay model.

### 2.8 Protein Degradation Pathway Analysis

To elucidate the pathway responsible for hypoxia‐induced FSP1 degradation, neutrophils were pre‐treated with the proteasome inhibitor MG132 (10 μM), the autophagy initiation inhibitor 3‐MA (5 mM), or the autophagosome‐lysosome fusion inhibitor BafA1 (100 nM) for 1 h prior to and during hypoxic exposure. Cells were harvested, and FSP1 protein levels were analyzed by Western blot. Restoration of FSP1 expression by a specific inhibitor suggested the involvement of the corresponding degradation pathway.

### 2.9 Western Blot and Antibodies

Cells or tissues were lysed in radioimmunoprecipitation assay (RIPA) buffer containing protease and phosphatase inhibitors. Protein concentrations were determined using a bicinchoninic acid (BCA) protein assay kit. Equal amounts of protein were separated by sodium dodecyl sulfate–polyacrylamide gel electrophoresis (SDS–PAGE), transferred to polyvinylidene difluoride (PVDF) membranes (Cat: #IPVH00010, Sigma‐Aldrich), and blocked with 5% non‐fat milk in Tris‐buffered saline with Tween 20 (TBST) buffer. Membranes were incubated overnight at 4°C with primary antibodies against FSP1 (Cat: #20886‐1‐AP, Proteintech), MARCH5 (Cat: #A30663, Bosterbio), MARCH6 (Cat: #DF9482, Affinity Biosciences), Flag‐tag proteins (Cat: #14793S, CST), HA‐tag‐proteins (Cat: #3724S, CST), Myc‐tag‐proteins (Cat: #2278S, CST), ubiquitin (Cat: #3936S, CST), β‐actin (Cat: #3700, CST). After washing, membranes were incubated with horseradish peroxidase (HRP)‐linked anti‐mouse IgG (Cat: #7076S, CST) or HRP‐linked anti‐rabbit IgG (Cat: #7074S, CST), and protein bands were visualized using enhanced chemiluminescence (ECL) reagents (Cat: #34098CN, Thermo Fisher Scientific). Densitometric analysis was performed using ImageJ software.

### 2.10 Co‐Immunoprecipitation (Co‐IP)

To examine the interaction between FSP1 and MARCH5, neutrophils were lysed in IP lysis buffer. Cell lysates (500 μg protein) were incubated with anti‐FSP1 antibody or control IgG overnight at 4°C with gentle rotation. Protein A/G agarose beads were added and incubated for an additional 2 h. The immunoprecipitates were washed extensively with lysis buffer, eluted by boiling in SDS sample buffer, and subjected to Western blot analysis using anti‐MARCH5 antibody. For reverse Co‐IP, lysates were immunoprecipitated with anti‐MARCH5 antibody and probed with anti‐FSP1 antibody.

### 2.11 Ubiquitination Assay

To detect FSP1 ubiquitination, neutrophils were transfected with plasmids expressing HA‐tagged ubiquitin (HA‐Ub) along with empty vector or MARCH5 overexpression constructs. Cells were treated with MG132 (10 μM) for 4 h to stabilize ubiquitinated proteins. Cell lysates were subjected to immunoprecipitation with anti‐FSP1 antibody, and ubiquitinated FSP1 was detected by Western blotting using anti‐HA antibody.

### 2.12 Gene Silencing and Overexpression

For gene silencing, neutrophils were transfected with small interfering RNA (siRNA) targeting *March5* (Cat: #sc‐149269, Santa Cruz Biotechnology) or a non‐targeting scrambled control siRNA (Cat: #sc‐37007, Santa Cruz Biotechnology) using Lipofectamine RNAiMAX(Cat: #13778150, Thermo Fisher Scientific). Knockdown efficiency was confirmed by Western blotting 48–72 h post‐transfection. For overexpression, cells were transfected with a pcDNA3.1 vector encoding full‐length MARCH5 cDNA or an empty control vector using Lipofectamine 3000 (Cat: #L3000015, Thermo Fisher Scientific), following the manufacturer's protocol.

### 2.13 Quantification of Serum Autoantibodies and Cytokines

Peripheral blood was collected via retro‐orbital bleeding at designated time points, and serum was separated by centrifugation and stored at −80°C until analysis. Anti‐double‐stranded DNA (dsDNA) antibody levels were measured using a commercially available mouse anti‐double‐stranded DNA antibody (IgG) enzyme‐linked immunosorbent assay (ELISA) kit (Cat: #CSB‐E11194m‐Q, CUSABIO). Serum concentrations of interferon‐alpha 2 (IFN‐α2) and complement component C3 were quantified using commercial ELISA kits (Cat. #ab211648, Abcam for IFN‐α2; Cat. #RK09112, Abclonal for C3) according to the manufacturers' protocols.

### 2.14 E3 Ligase Prediction Using UbiBrowser

Potential E3 ubiquitin ligases for FSP1 were predicted using the UbiBrowser database (http://ubibrowser.ncpsb.org.cn), a bioinformatics tool for predicting human ubiquitin ligase‐substrate relationships. FSP1 was input as the query substrate, and the top‐ranked candidates were filtered based on confidence scores and literature relevance. MARCH5 and MARCH6 were identified as the highest‐confidence candidate E3 ligases for subsequent experimental validation.

### 2.15 Statistical Analysis

Data are presented as mean ± standard deviation (SD). Statistical analyses were performed using GraphPad Prism (version 10.6.0). Comparisons between two independent groups were performed using an unpaired two‐tailed Student's *t*‐test. Survival curves were generated using the Kaplan–Meier method and compared using the log‐rank test. For animal experiments, each individual mouse was considered an experimental unit for animal‐level outcomes. For cell‐based assays, each independently prepared bone marrow‐derived neutrophil preparation or independently performed cell‐based experiment was considered one biological replicate. Technical replicate wells within the same experiment were averaged before statistical analysis and were not treated as independent observations. The exact sample size, statistical test, and definition of *n* for each analysis are provided in the corresponding figure legends. All statistical tests were two‐sided, and *p* < 0.05 was considered statistically significant.

### 2.16 Study Design, Sample Size, and Bias Control

For the in vivo experiments, mice were assigned to normoxic (21% O_2_) or hypoxic (11% O_2_) conditions, as described in the corresponding Methods sections and figure legends. In experiments involving neutrophil‐specific March5 deletion, *March* ^fl/fl^Ly6g‐Cre^+^ mice were compared with *March* ^fl/fl^Ly6g‐Cre^−^ mice under the indicated experimental conditions. For animal‐level outcomes, each individual mouse was considered an experimental unit. For cell‐based experiments, each independently prepared neutrophil preparation was considered an experimental unit. Technical replicate wells from the same independent experiment were averaged before statistical analysis and were not treated as independent biological replicates. No a priori statistical power calculation was performed to determine sample sizes. Group sizes were determined with reference to previously published studies [8, 23]. A single primary outcome measure was not prespecified for sample‐size determination. The outcomes assessed in the in vivo SLE experiments included survival, serum anti‐dsDNA antibody levels, IFN‐α2 and C3 levels, spleen measurements, and renal histopathology. Mechanistic experiments assessed neutrophil ferroptosis‐associated outcomes, including cell viability, lipid reactive oxygen species (lipid ROS), 4‐hydroxynonenal (4‐HNE), FSP1 expression and protein stability, MARCH5–FSP1 interaction, and FSP1 ubiquitination. The exact sample size for each analysis is reported in the corresponding figure legends. All animals allocated to the experiments were included in the analyses, and no animals were excluded. Mice were randomly assigned to normoxic or hypoxic conditions by drawing lots. The order of treatments and measurements and cage positions were randomized to minimize potential confounding effects. Data collection and analysis were not performed blind to the experimental conditions.

## 3 Results

### 3.1 Chronic Hypoxia Exacerbates SLE Pathology

Due to vascular dysfunction and mitochondrial abnormalities, SLE kidneys frequently exhibit hypoxic features [24, 25]. To determine the impact of hypoxia on SLE progression, we housed SLE mice under hypoxic (11% O_2_) or normoxic (21% O_2_) conditions (Figure 1A). Compared with normoxic controls, hypoxia‐exposed mice exhibited reduced survival, increased serum dsDNA and IFN‐α2 levels, and decreased complement C3 levels (Figure 1B–E). Consistently, hypoxic mice displayed aggravated splenomegaly and renal pathological injury (Figure 1F,G). These findings demonstrate that chronic hypoxia exacerbates systemic inflammation and organ damage in SLE.

**Figure 1:**
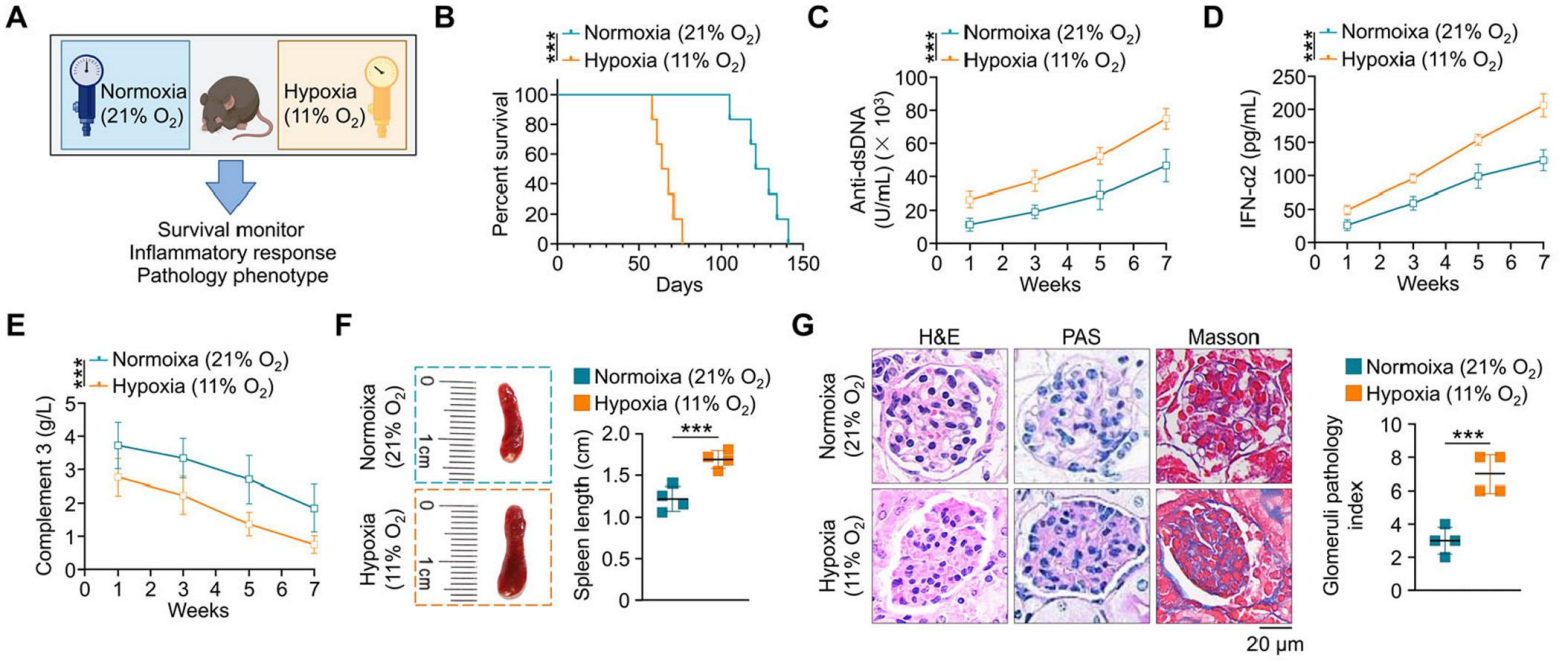
Hypoxia exacerbates the SLE‐associated pathology. (A) The schematic diagram shows that mice with SLE were raised in the normoxia (21% O_2_) or hypoxia (11% O_2_) conditions. (B–E) The survival (B), anti‐dsDNA levels (C), IFN‐α2 (D), and Complement 3 (E) in serum from SLE mice raised in the normoxia (21% O_2_) or hypoxia (11% O_2_) conditions were measured (*n* = 4 mice per group). (F and G) SLE mice raised in the normoxia (21% O_2_) or hypoxia (11% O_2_) conditions were sacrificed, and the spleen and kidney were isolated. The spleen volume and length were compared (F), and the kidneys were subjected to H&E, PAS, and Masson's trichrome staining (G) (*n* = 4 mice per group). All error bars represent the mean ± SD. *p* values were calculated using a two‐tailed Student's *t*‐test. ***p < 0.001.

### 3.2 Hypoxia Impairs FSP1‐Dependent Ferroptosis Defense in Neutrophils

Neutrophils constitute the primary leukocyte subset in peripheral blood and are recognized as key mediators in a range of autoimmune pathologies, including SLE. To investigate whether hypoxia contributes to the development and progression of SLE through neutrophil‐mediated mechanisms, we isolated neutrophils (Supporting Information S1: Figure S1A,B) and used pimonidazole staining to assess hypoxia in the spleen and kidneys of SLE‐model mice, with a focus on neutrophils (Figure 2A). The results revealed that, compared to those from healthy control mice, neutrophils isolated from the spleen and kidney of SLE mice exhibited increased hypoxic signals (Figure 2B). Hypoxia can regulate ferroptosis in a context‐dependent manner through distinct molecular pathways, including HIF‐dependent signaling. For example, HIF activation enhances the transcription of *SLC1A1*, a component of the cystine/glutamate antiporter system, thereby promoting cystine uptake and increasing cellular resistance to ferroptosis [26, 27, 28]. In addition, neutrophil ferroptosis has been implicated in the pathogenesis and disease manifestations of autoimmune disorders [8]. To this end, we further quantified the levels of lipid reactive oxygen species (Lipid ROS) and 4‐hydroxynonenal (4‐HNE), two key hallmarks of ferroptosis, in neutrophils isolated from the spleen and kidney of mice housed under either normoxic or hypoxic conditions. The results demonstrated that, even under normoxia, neutrophils from the spleen and kidney of SLE mice already exhibited significantly elevated levels of Lipid ROS and 4‐HNE compared to those from healthy controls (Figure 2C). Moreover, housing under hypoxic conditions further upregulated these two ferroptotic markers in neutrophils residing in the aforementioned organs of SLE mice (Figure 2C). To further validate the promoting effect of a hypoxic environment on the ferroptotic process in neutrophils, we treated mouse bone marrow‐derived neutrophils with the ferroptosis inducer Erastin, which acts by blocking cystine uptake and glutathione synthesis. The experimental results demonstrated that hypoxic conditions markedly exacerbated Erastin‐triggered ferroptotic effects in neutrophils; more importantly, this exacerbation could be effectively reversed by the ferroptosis‐specific inhibitor Ferrostatin‐1 (Fer‐1, a lipid peroxidation blocker) (Figure 2D).

**Figure 2:**
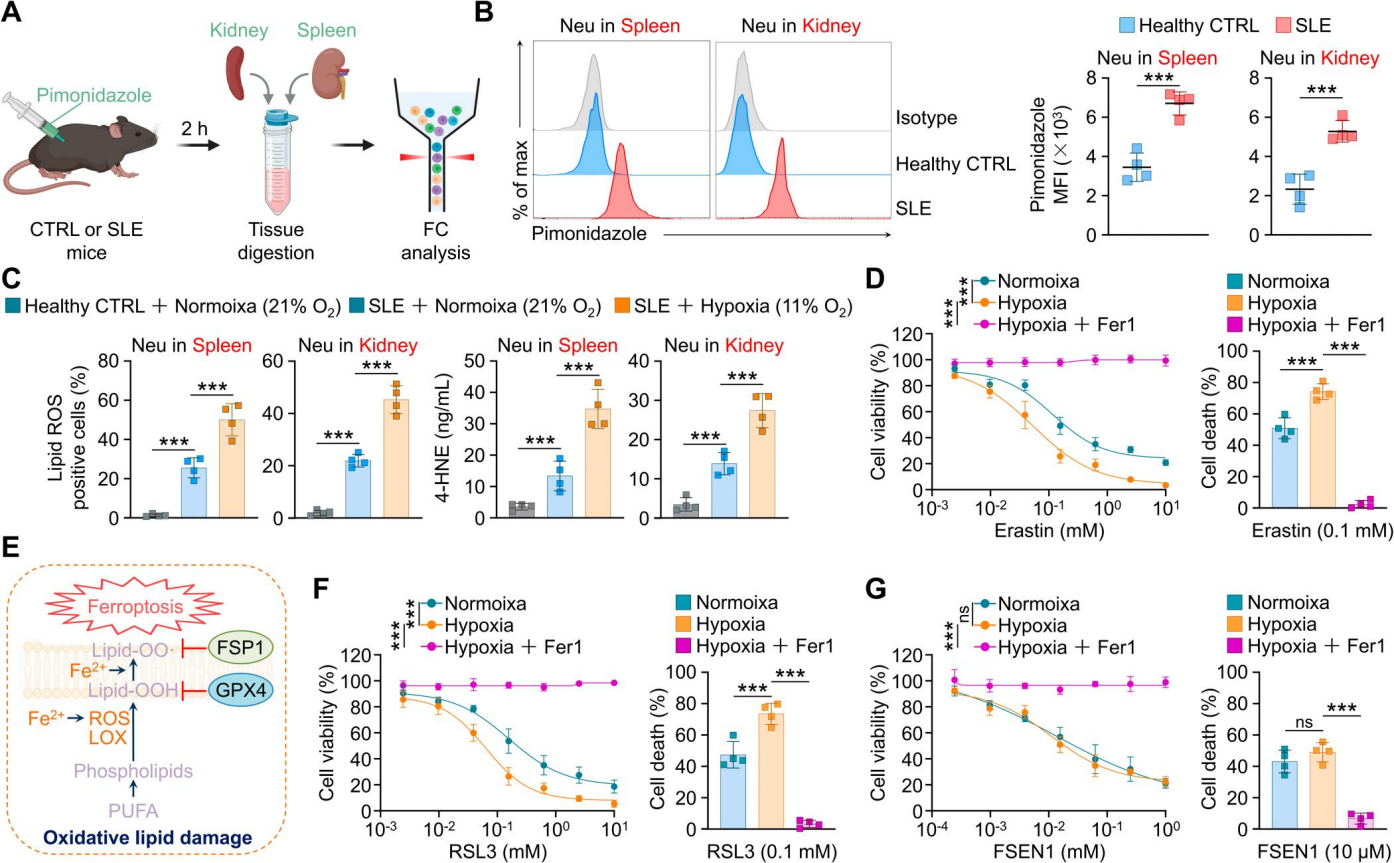
Hypoxia promotes FSP1‐dependent ferroptosis in neutrophils. (A–C) The schematic diagram (A) that mice were intraperitoneally injected with Pimonidazole 2 h before sacrifice, and the spleen and kidneys were collected, neutrophils were sorted, and the fluorescence intensity of Pimonidazole in neutrophils was detected using flow cytometry (B), and the lipid ros and 4‐HNE were detected (C) (*n* = 4 mice per group). (D) Bone marrow‐derived primary neutrophils were cultured under normoxia or hypoxia with or without treatment with Erastin or Fer‐1, and the rate of cell death was measured using the CCK‐8 assay (*n* = 4 biological experiments). (E) The schematic diagram of ferroptosis resistance involving GPX4 and FSP1. (F and G) Bone marrow‐derived primary neutrophils were cultured under normoxia or hypoxia with or without treatment with RSL3, FSEN1, or Fer‐1, and the rate of cell death was measured using the CCK‐8 assay (*n* = 4 biological experiments). All error bars represent the mean ± SD. *p* values were calculated using a two‐tailed Student's *t*‐test. ***p < 0.001; ns, not significant.

In the ferroptosis defense machinery, GPX4 serves as the canonical suppressor, reducing lipid peroxides through the utilization of glutathione. In contrast, FSP1 functions independently of GPX4, scavenging lipid radicals by reducing coenzyme Q10 to its antioxidant form (Figure 2E). To investigate whether a hypoxic environment accelerates neutrophil ferroptosis by modulating the ferroptosis defense system, we employed RSL3 and FSEN1 to inhibit GPX4‐ and FSP1‐mediated ferroptosis defense, respectively. We found that hypoxic conditions markedly potentiated RSL3‐induced ferroptotic cell death, whereas no significant additional effect was observed upon FSEN1 treatment (Figure 2F,G). Furthermore, ferrostatin‐1 (Fer‐1) effectively suppressed ferroptotic cell death under both conditions (Figure 2F,G). Collectively, these findings suggest that hypoxia may promote neutrophil ferroptosis by compromising FSP1‐mediated ferroptosis defense.

### 3.3 Hypoxia Induces MARCH5‐Mediated Ubiquitination and Degradation of FSP1

To determine whether hypoxia directly regulates FSP1 expression, we subjected neutrophils to hypoxic intervention and subsequently assessed the intracellular levels of *Aifm2* mRNA, which encodes FSP1, and FSP1 protein. The results showed that hypoxic treatment markedly shortened the half‐life of FSP1 protein (Figure 3A), without significantly altering *Aifm2* mRNA levels (Figure 3B). Given that intracellular protein degradation primarily relies on the proteasome and autophagy‐lysosome pathways, we further employed specific inhibitors targeting these respective routes to dissect the underlying mechanism. The experimental data revealed that only the proteasome inhibitor MG132 effectively reversed the hypoxia‐induced decline in FSP1 protein content; in contrast, autophagy‐lysosome pathway blockers, such as 3‐MA and BafA1, exhibited no discernible protective effect (Figure 3C). Collectively, these findings indicate that hypoxia‐induced FSP1 degradation is predominantly mediated by the ubiquitin‐proteasome system.

**Figure 3:**
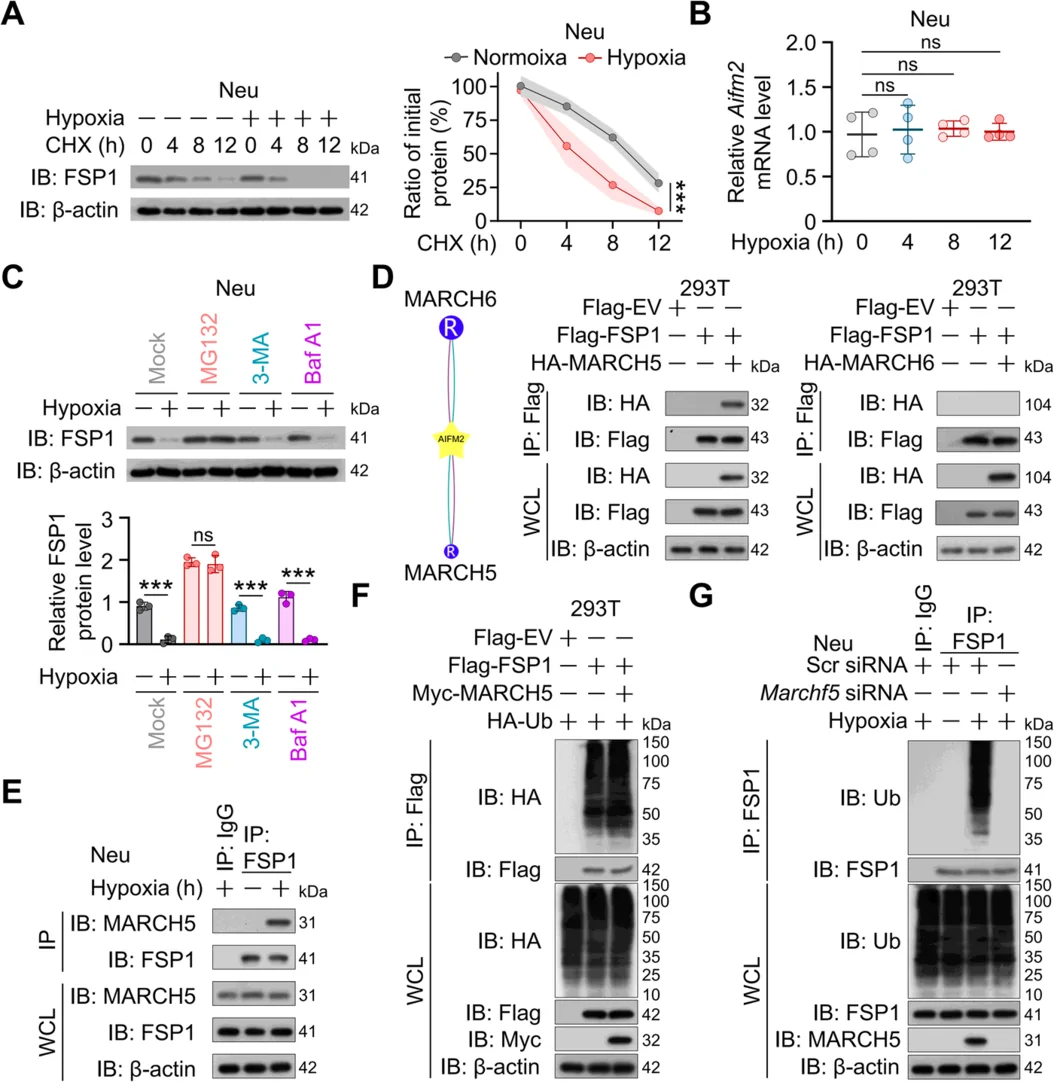
Hypoxia‐driven ferroptosis is MARCH5‐dependent. (A) Bone marrow‐derived primary neutrophils were treated with CHX for the indicated time, together with or without hypoxia treatment. Relative intensity of FSP1 to β‐tubulin was analyzed (*n* = 4 independent experiments). (B) The mRNA expression levels of *Aifm2* gene in hypoxia‐treated bone marrow‐derived primary neutrophils were measured using qPCR (*n* = 4 independent experiments). (C) Immunoblotting analysis of hypoxia‐treated bone marrow‐derived primary neutrophils treated with MG132 (10 μM), 3‐MA (5 mM), or Baf A1 (0.1 μM) for 6 h, and the relative protein level was quantified (*n* = 4 independent experiments). (D) The potential E3 ligases were predicted using UbiBrowser database, and the co‐immunoprecipitation (co‐IP; with anti‐FLAG) and immunoblotting analysis using protein lysates of HEK293T cells transfected with the indicated plasmids. WCL, whole cell lysates. (E) Co‐IP and immunoblotting analysis using protein lysates of bone marrow‐derived primary neutrophils treated with hypoxia. (F) HEK293T cells were transfected with FLAG‐FSP1 and HA‐tagged WT ubiquitin (HA‐Ub), together with MYC‐EV or MYC‐MARCH5 for 24 h, followed by treatment with MG132 for 6 h, followed by co‐immunoprecipitated with anti‐Flag beads and immunoblotted with anti‐HA antibody. (G) Bone marrow‐derived primary neutrophils were transfected with Scr siRNA or MARCH5‐specific siRNA for 24 h, followed by transfected with FLAG‐EV or FLAG‐FSP1 for 24 h; protein lysates were harvested after hypoxia treatment for 12 h and MG132 treatment for 6 h for IP (with anti‐FSP1) and immunoblotting analysis using indicated antibodies. Molecular weight markers are indicated on the left (kDa). All error bars represent the mean ± SD. *p* values were calculated using a two‐tailed Student's *t*‐test. ***p < 0.001; ns, not significant.

Using the UbiBrowser database for bioinformatic prediction, we identified MARCH5 and MARCH6 as potential E3 ubiquitin ligases for FSP1 (Figure 3D). Subsequent co‐immunoprecipitation analysis further confirmed that FSP1 specifically interacts with MARCH5 (Figure 3D). Notably, the interaction intensity between FSP1 and MARCH5 was markedly enhanced in neutrophils subjected to hypoxic stimulation (Figure 3E). Consistent with this observation, overexpression of MARCH5 significantly increased the level of FSP1 ubiquitination (Figure 3F), whereas knockdown of MARCH5 effectively blocked hypoxia‐triggered FSP1 ubiquitination (Figure 3G). Collectively, this study reveals that hypoxia accelerates FSP1 degradation via the MARCH5‐mediated ubiquitin‐proteasome pathway.

### 3.4 MARCH5 Mediates Hypoxia‐Induced Neutrophil Ferroptosis Through FSP1 Suppression

To validate the functional role of MARCH5 in hypoxia‐mediated ferroptosis, we assessed the effect of MARCH5 knockdown on Erastin‐ and FSEN1‐induced ferroptosis in neutrophils. The results demonstrated that MARCH5 silencing reduced Erastin‐induced ferroptotic cell death under hypoxic conditions and concomitantly reduced intracellular 4‐HNE levels (Figure 4A–C). However, when FSEN1 was used to block FSP1 enzymatic activity, silencing MARCH5 failed to eliminate the promoting effect of hypoxia on ferroptosis (Figure 4D–F). These results indicate that the ferroptosis‐promoting function of MARCH5 is mediated through FSP1 suppression.

**Figure 4:**
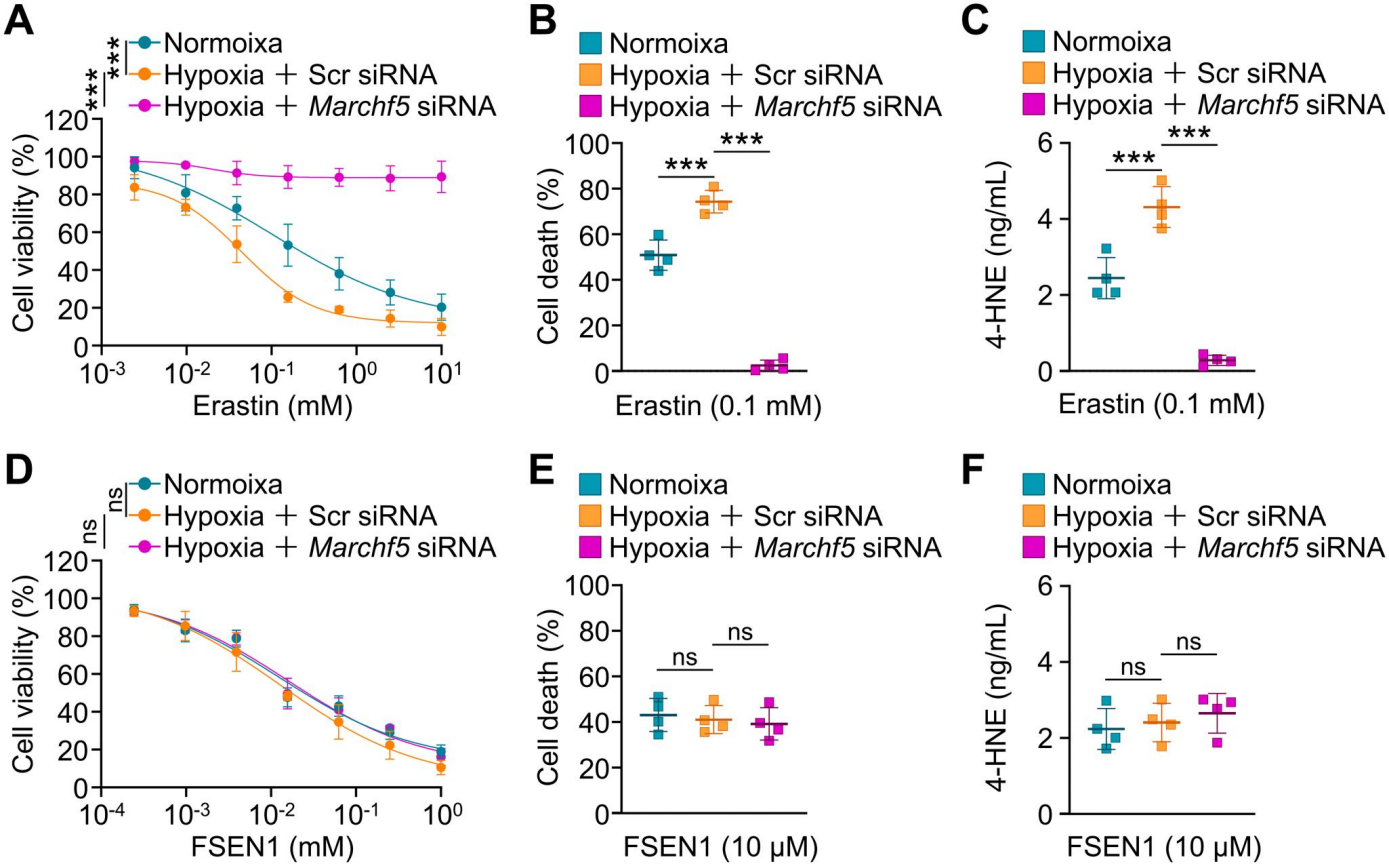
Hypoxia‐driven ferroptosis is MARCH5‐dependent. (A–F) Bone marrow‐derived primary neutrophils were transfected with Scr siRNA or MARCH5‐specific siRNA for 24 h, and then cultured under normoxia or hypoxia with or without treatment with Erastin (A–C) or FSEN1 (D–F), and the rate of cell death was measured using the CCK‐8 assay (A, D), and the lipid ros (B, E) and 4‐HNE were detected (C, F) (*n* = 4 independent experiments). All error bars represent the mean ± s.d. *p* values were calculated using a two‐tailed Student's *t*‐test. ***p < 0.001; ns, not significant.

### 3.5 Neutrophil MARCH5 Promotes Hypoxia‐Associated SLE Progression

To elucidate the pathophysiological significance of MARCH5‐promoted neutrophil ferroptosis under hypoxic conditions, we generated a neutrophil‐specific MARCH5 knockout mouse model (Figure 5A). The experimental results demonstrated that, compared with wild‐type controls, neutrophil‐specific MARCH5 deficiency significantly improved the survival rate of SLE mice (Figure 5B), reduced the titers of double‐stranded DNA antibodies and interferon‐α2 in peripheral blood (Figure 5C,D), and was accompanied by a recovery of serum complement C3 levels (Figure 5E). Subsequent histopathological analysis further revealed that neutrophil‐specific MARCH5 knockout also effectively ameliorated splenomegaly and renal tissue injury in SLE mice (Figure 5F,G). Collectively, these findings demonstrate that MARCH5 plays a critical pathogenic role in SLE progression by mediating hypoxia‐induced neutrophil ferroptosis.

**Figure 5:**
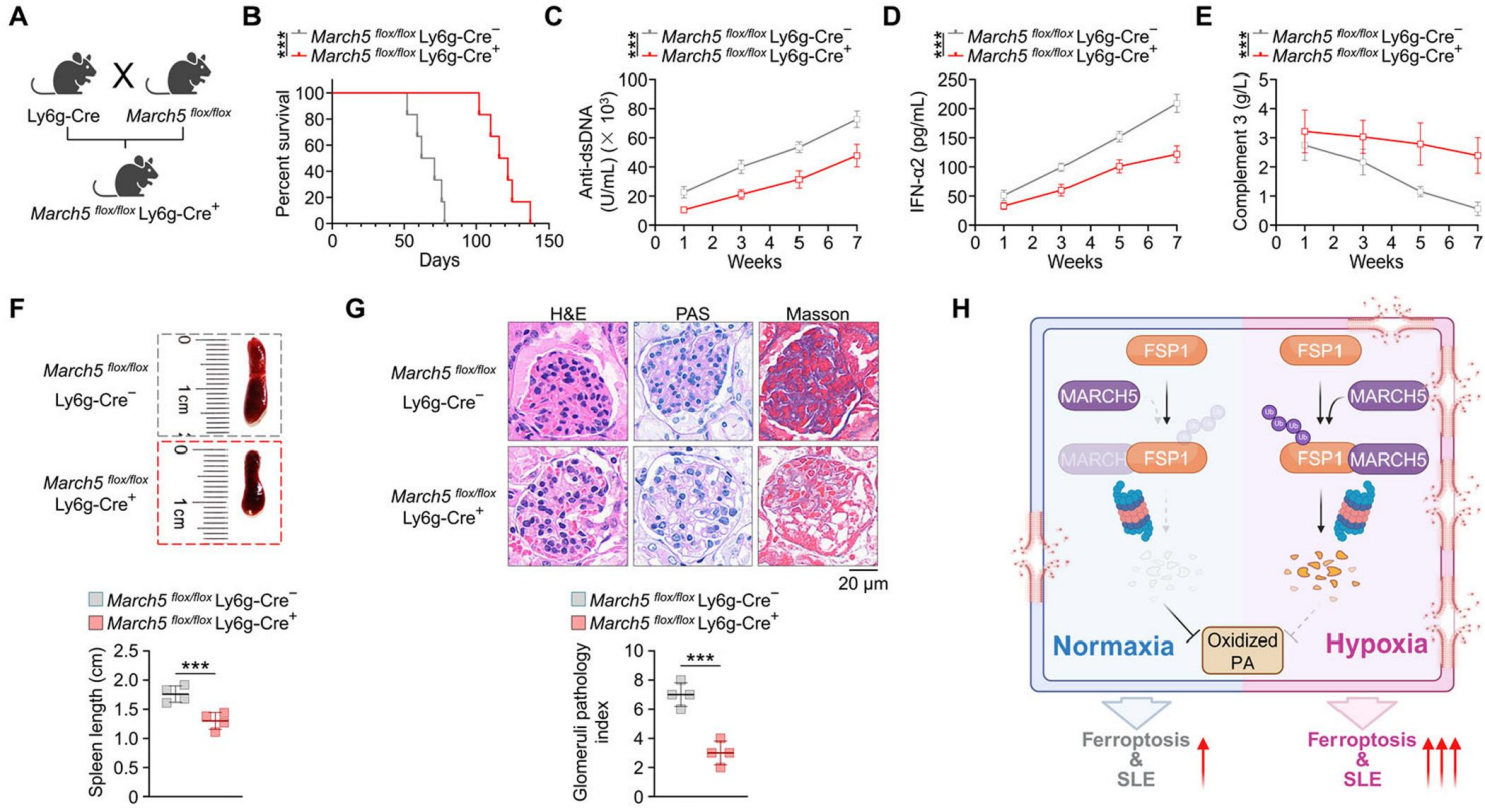
MARCH5 in neutrophils drives hypoxia‐associated SLE pathology. (A) The schematic diagram for *March5* ^fl/fl^Ly6g‐Cre^+^ mice generation. (B–E) The survival (B), anti‐dsDNA levels (C), IFN‐α2 (D), and Complement 3 (E) in serum from indicated SLE mice raised in the hypoxia (11% O_2_) conditions were measured (*n* = 4 mice per group). (F and G) The indicated SLE mice raised in the hypoxia (11% O_2_) conditions were sacrificed, and the spleen and kidney were isolated. The spleen volume and length were compared (F), and the kidneys were subjected to H&E, PAS and Masson's trichrome staining (G) (*n* = 4 mice per group). (H) The graphical abstract for this study. All error bars represent the mean ± SD. *p* values were calculated using a two‐tailed Student's *t*‐test. ***p < 0.001.

## 4 Discussion

In this study, we identify a previously unrecognized pathogenic axis involving hypoxia, the E3 ubiquitin ligase MARCH5, and the ferroptosis defense factor FSP1 in SLE progression. Although the canonical ferroptosis regulator GPX4 has been implicated in SLE pathogenesis, the contribution of the parallel FSP1–CoQ10–NAD(P)H pathway to autoimmune disease remains poorly understood. Our findings demonstrate that hypoxic microenvironments, which frequently arise in injured SLE tissues, preferentially promote neutrophil ferroptosis by inducing MARCH5‐mediated ubiquitination and proteasomal degradation of FSP1. This mechanism amplifies systemic inflammation and organ damage, establishing the hypoxia–MARCH5–FSP1 axis as a determinant of SLE severity (Figure 5H).

Our initial observations indicate that chronic hypoxia reduces survival and pathological indices in lupus‐prone mice, in alignment with clinical data showing that tissue hypoxia correlates with disease activity in lupus nephritis. While previous studies have linked hypoxia to altered immune metabolism and IFN‐I signaling [29, 30, 31], our study identifies neutrophil ferroptosis as a central cellular consequence of this environmental stressor. Neutrophils, known for their high glycolytic activity, are particularly sensitive to changes in oxygen tension [32, 33, 34]. The increased lipid peroxidation and elevated 4‐HNE levels observed in hypoxic SLE neutrophils highlight a state of heightened ferroptotic susceptibility in vivo. Importantly, hypoxia markedly enhanced RSL3‐induced ferroptosis but did not significantly increase FSEN1‐induced ferroptosis, suggesting that hypoxia preferentially compromises FSP1‐mediated ferroptosis defense.

A critical breakthrough of this study is the identification of the molecular machinery responsible for hypoxia‐induced FSP1 degradation. We demonstrate that hypoxia does not alter *Aifm2* mRNA expression but instead reduces FSP1 protein stability through the ubiquitin‐proteasome system. Through database screening and rigorous biochemical validation, we identified MARCH5, a mitochondrial outer membrane E3 ubiquitin ligase [35], as the specific enzyme responsible for targeting FSP1 for degradation under low oxygen conditions. Traditionally, MARCH5 is known for regulating mitochondrial dynamics and mitophagy by ubiquitinating proteins such as SLC25A46 and FUNDC1 [36, 37]. Our work extends the functional repertoire of MARCH5 to include the regulation of ferroptosis sensitivity in immune cells. The enhanced interaction between MARCH5 and FSP1 under hypoxic conditions may reflect stress‐induced activation, redistribution, or conformational changes of MARCH5 or FSP1. The rescue of FSP1 expression and inhibition of ferroptosis upon silencing *March5* provides strong evidence for the role of MARCH5 in regulating FSP1 stability.

Furthermore, the generation and analysis of neutrophil‐specific *March5* knockout mice provided critical in vivo validation of the pathophysiological relevance of this mechanism. The observed attenuation of dsDNA titers, inflammatory cytokines, and renal pathology in knockout mice under hypoxic conditions firmly establishes that MARCH5 activity in neutrophils exacerbates SLE. These findings suggest a potential therapeutic vulnerability: stabilizing FSP1 protein levels by inhibiting MARCH5 function, or by developing FSP1 mimetics, could specifically restore ferroptosis defense in inflamed tissues without broadly disrupting physiological cell death pathways necessary for immune homeostasis.

While this study advances our understanding of ferroptosis regulation in SLE, there are several limitations that should be acknowledged. First, our in vivo model primarily relies on TLR7/8 agonist induction, which models an IFN‐driven lupus signature. It remains to be determined whether the hypoxia‐MARCH5‐FSP1 axis is similarly dominant in spontaneous lupus models, such as MRL/*lpr*, which feature more pronounced T cell and B cell dysregulation. Second, although we employed pimonidazole staining to confirm neutrophil hypoxia, the precise oxygen gradient in situ within inflamed tissues, such as the kidney or spleen, remains to be quantified. Additionally, while MARCH5 is a mitochondrial ligase and FSP1 localizes to the plasma membrane and lipid droplets, the spatiotemporal dynamics of their interaction during hypoxia have not been fully elucidated. It is conceivable that hypoxia‐induced reactive oxygen species or mitochondrial stress signals precede the MARCH5‐FSP1 interaction, a hypothesis that warrants future investigation.

Several limitations of this study should be acknowledged. Although our findings indicate that hypoxia preferentially impairs FSP1‐dependent, rather than GPX4‐dependent, ferroptosis defense in neutrophils, the basis for this selectivity remains unresolved. FSP1 and GPX4 suppress lipid peroxidation through distinct but complementary mechanisms: FSP1 supports CoQ reduction in an NAD(P)H‐dependent manner, whereas GPX4 reduces phospholipid hydroperoxides using GSH. Hypoxia could therefore affect these pathways differently by altering their respective protein stability, localization, cofactor availability, or enzymatic activity. In particular, it remains unclear whether the observed selectivity reflects the preferential regulation of FSP1 by MARCH5, differences in the metabolic requirements of the two defense systems, or both. We did not comprehensively assess pathway activity or redox status, including CoQ/CoQH_2_, NAD(P)H/NAD(P)^+^, GSH/GSSG, or lipid‐peroxidation flux. Future studies should define how hypoxia regulates these components and determine whether the same pathway selectivity occurs across different neutrophil states, cell types, and SLE disease settings.

Moreover, the therapeutic implications of the MARCH5–FSP1 axis also require further investigation. MARCH5 regulates mitochondrial protein turnover and homeostasis; therefore, systemic MARCH5 inhibition could affect substrates beyond FSP1, disturb mitochondrial function, or alter the activity and survival of other immune and tissue cells. Although stabilizing FSP1 may offer a more focused approach to preserving ferroptosis defense, the pharmacological feasibility of doing so and the consequences of increasing FSP1 activity in other cell types remain unknown. Similarly, developing MARCH5‐selective inhibitors or selectively disrupting the MARCH5–FSP1 interaction will require evidence that the intervention can reduce pathological neutrophil ferroptosis without broadly impairing mitochondrial homeostasis. Future work should test these strategies in relevant in vivo models and assess their cell specificity, effects on mitochondrial function and immune responses, and dose‐dependent safety.

## 5 Conclusion

In conclusion, our study identifies hypoxia‐induced MARCH5‐mediated degradation of FSP1 as a novel and critical pathway that promotes neutrophil ferroptosis and exacerbates the progression of SLE. This work not only enhances our understanding of why the FSP1 defense system fails in chronic inflammatory diseases but also nominates the MARCH5‐FSP1 interface as a promising target for therapeutic intervention aimed at mitigating tissue damage in lupus and potentially other ferroptosis‐associated autoimmune conditions. Future studies should explore the development of small‐molecule inhibitors targeting the MARCH5‐FSP1 interaction as a strategy to enhance cellular resilience against ferroptotic death in the hypoxic inflammatory milieu of SLE.

## Author Contributions

Zhiwei Liu conceived the study, Jing Zhang conducted cell and molecular biology experiments, drafted the main manuscript, analyzed experimental data, and summarized the findings. Jing Zhang and Fulin Cai prepared the figures. Zhiwei Liu and Jiansen Lu provided guidance on experimental design and manuscript writing. All authors made substantial contributions to the work, reviewed and revised the manuscript editorially, and approved the final version. Each author agrees to be accountable for all aspects of the work.

## Ethics Statement

All experimental procedures involving animals were approved by the ethics committee of Sir Run Run Shaw Hospital. All animal studies were conducted according to the Association for the Assessment and Accreditation of Laboratory Animal Care and the Institutional Animal Care and Use Committee guidelines. All experiments were carried out in accordance with approved guidelines.

## Conflicts of Interest

The authors declare no conflicts of interest.

## Supporting information


**Figure S1:** Hypoxia promotes FSP1‐dependent ferroptosis in neutrophils.(A) Representative flow cytometry gating strategy for identification of purified bone marrow‐derived neutrophils. Neutrophils were defined as the Ly6G⁺CD11b⁺ population after density gradient centrifugation. Representative plots showing the Ly6G⁺CD11b⁺ population are presented, with the percentage of gated neutrophils indicated. (B) Assessment of neutrophil viability after isolation by 7‐AAD staining. Representative flow cytometry profiles showing the percentage of 7‐AAD‐negative viable neutrophils are presented.

## Data Availability

The data that support the findings of this study are available from the corresponding author upon reasonable request.
