# Supplementary material for: MARCH5 Links Hypoxia to Neutrophil Ferroptosis in SLE via FSP1 Ubiquitination
Source: Immun Inflamm Dis. 2026 Nov 10;14(11):e70583. doi: 10.1002/iid3.70583 (PMC13749613; doi:10.1002/iid3.70583)
Supplement: Supplementary file 1 [file IID3-14-e70583-s001.docx]

**Figure S1 Hypoxia promotes FSP1-dependent ferroptosis in neutrophils.**

**(A)** Representative flow cytometry gating strategy for identification of purified bone marrow-derived neutrophils. Neutrophils were defined as the Ly6G⁺CD11b⁺ population after density gradient centrifugation. Representative plots showing the Ly6G⁺CD11b⁺ population are presented, with the percentage of gated neutrophils indicated. **(B)** Assessment of neutrophil viability after isolation by 7-AAD staining. Representative flow cytometry profiles showing the percentage of 7-AAD-negative viable neutrophils are presented.
